# EMP3 negatively modulates breast cancer cell DNA replication, DNA damage repair, and stem-like properties

**DOI:** 10.1038/s41419-021-04140-6

**Published:** 2021-09-12

**Authors:** Kailing Zhou, Yu Sun, Dan Dong, Chenghai Zhao, Wei Wang

**Affiliations:** Department of Pathophysiology, College of Basic Medical Science, China Medical University, Shenyang, China

**Keywords:** Breast cancer, Cancer therapeutic resistance

## Abstract

Enhanced DNA damage repair capacity attenuates cell killing of DNA-damaging chemotherapeutic agents. In silico analysis showed that epithelial membrane protein 3 (EMP3) is associated with favorable survival, and negatively regulates cell cycle S-phase. Consistently, loss and gain of function studies demonstrated that EMP3 inhibits breast cancer cell S-phage entry, DNA replication, DNA damage repair, and stem-like properties. Moreover, EMP3 blocks Akt-mTOR signaling activation and induces autophagy. EMP3 negatively modulates BRCA1 and RAD51 expression, indicating EMP3 suppresses homologous recombination repair of DNA double-strand breaks. Accordingly, EMP3 sensitizes breast cancer cells to the DNA-damaging drug Adriamycin. EMP3 downregulates YTHDC1, a RNA-binding protein involved in m6a modification, which at least in part mediates the effects of EMP3 on breast cancer cells. Taken together, these data indicate that EMP3 is a putative tumor suppressor in breast cancer, and EMP3 downregulation may be responsible for breast cancer chemoresistance.

## Introduction

Chemotherapy currently remains a conventional treatment for breast cancer. However, breast cancers usually develop chemoresistance. An increase in DNA damage repair capacity endows cancer cells with insensitivity to DNA-damaging anti-cancer drugs. Homologous recombination repair of DNA double-strand breaks has been demonstrated to be responsible for cancer chemoresistance [1–3]. BRCA1 and RAD51 are two key factors involved in homologous recombination repair. Upregulation of BRCA1 and RAD51 impairs the killing effect of DNA-damaging agents on breast cancer cells [4–6]. DNA replication is closely associated with DNA damage repair. Homologous recombination is active in the replication phase of the cell cycle [7]. Actually, homologous recombination repair and DNA replication are usually modulated by the same factor or signaling pathway [8–10].

During breast cancer development, a small section of malignant epithelial cells exhibits some stem-like properties, including chemoresistance. These stem-like cells express several markers such as ALDH1, CD44, EPCAM, and CD133 [11, 12]. The generation of stem-like cancer cells is related to the upregulation of transcription factors SOX2, POU5F1, and NANOG [13, 14]. Stem-like cancer cells have increased DNA damage repair capacity in response to DNA-damaging agents, thereby possessing chemoresistance [15]. It has been reported that genes regulating homologous recombination and cell proliferation are upregulated in human-induced pluripotent stem (hiPS) cells, which exhibit a striking capacity to repair damaged DNA [16].

Epithelial membrane protein 3 (EMP3), a member of the PMP22 gene family, is involved in human malignant tumors. It functions as a tumor suppressor in neuroblastoma [17]. Ectopic expression of EMP3 suppressed neuroblastoma cell growth in vitro and in vivo. Moreover, EMP3 gene hypermethylation which causes EMP3 downregulation was shown associated with poor survival. EMP3 also plays a putative tumor-suppressing role in esophageal squamous cell carcinoma and gallbladder cancer [18, 19]. To the contrary, EMP3 promotes cell growth and migration in upper urinary tract urothelial carcinoma, hepatocellular carcinoma, and gastric cancer [20–22]. EMP3 was also found to induce certain breast cancer cell growth [23]. These findings indicate that the role of EMP3 in human tumors is tumor-specific or cell-specific. Here EMP3 was demonstrated as a tumor suppressor in breast cancer and a target to overcome chemoresistance, as a result of the negative modulation of DNA replication, DNA damage repair, and stemness.

## Results

### In silico analysis suggests EMP3 as a tumor suppressor in breast cancer

**Fig. 1:**
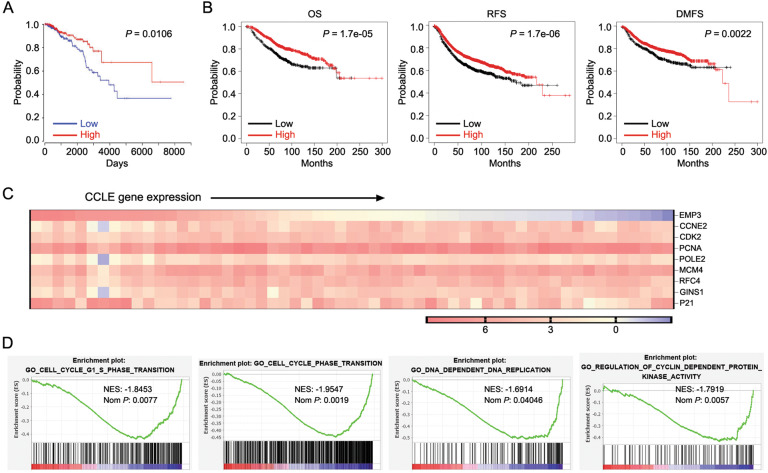
In silico analysis suggests EMP3 as a tumor suppressor in breast cancer. **A** The association of EMP3 with OS was analyzed in the TCGA database. **B** The association of EMP3 with OS, RFS, and DMFS was analyzed in Kaplan–Meier plotter website. **C** A heat map was generated from the CCLE database demonstrating the correlation of EMP3 with factors related to the S-phase. **D** Go analysis of the gene pathways differentially expressed between EMP3-high and EMP3-low breast cancer samples in the TCGA database was performed. Four representative GSEA-enrichment plots were shown.

To explore whether EMP3 plays a role in breast cancer, the association of EMP3 with the survival of breast cancer patients was analyzed in the TCGA database and Kaplan–Meier plotter website. Higher EMP3 expression was shown to be correlated with longer overall survival (OS), recurrence-free survival (RFS), and distal metastasis-free survival (DMFS) (Fig. 1A, B). Notably, EMP3 was shown to be associated with a favorable prognosis in four subtypes of breast cancer (Supplementary Fig. 1). To further deduce EMP3-associated phenotype, the correlation of EMP3 with other genes was analyzed in the CCLE database including a series of breast cancer cell lines. EMP3 was revealed to be negatively correlated with a panel of genes related to cell cycle S-phase such as CCNE2, CDK2, PCNA, RFC4, POEI2, MCM4, and GINS1 (Fig. 1C). Consistent with this finding, the Gene Set Enrichment Analysis (GSEA) of the TCGA database demonstrated EMP3 to be negatively related to cell cycle transition and DNA replication (Fig. 1D). Taken together, these in silico analyses suggest that EMP3 functions as a tumor suppressor in breast cancer.

### EMP3 blocks S-phase entry and DNA replication

**Fig. 2:**
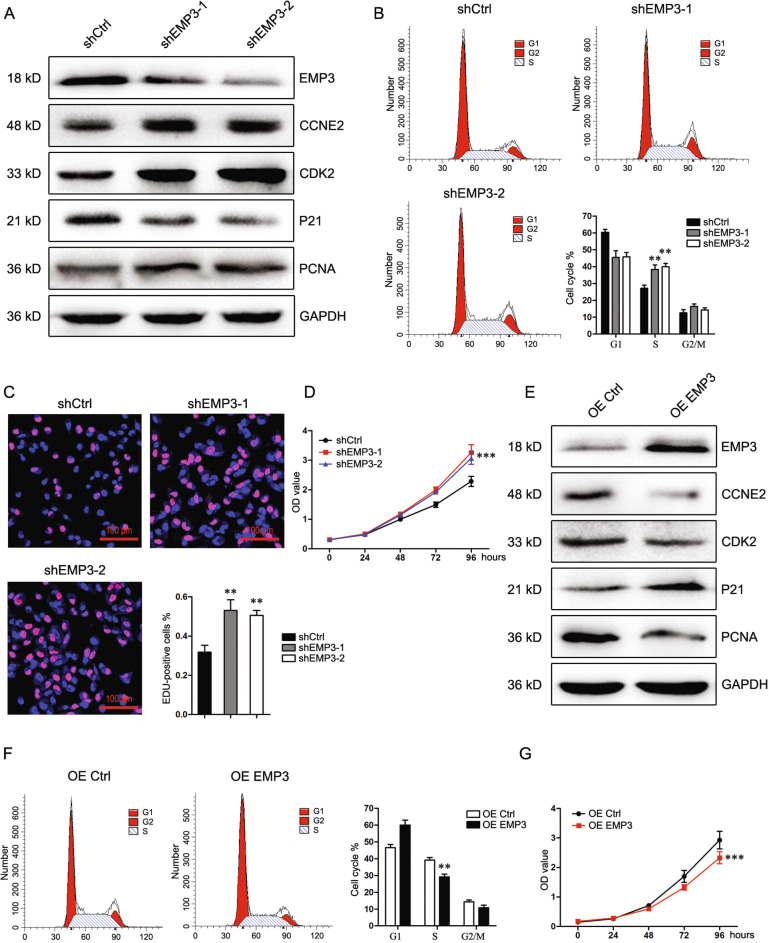
EMP3 blocks S-phase entry and DNA replication. **A** EMP3, CCNE2, CDK2, P21, and PCNA expression in MDA-MB-231 cells was detected by western blot. **B** Cell cycle of MDA-MB-231 cells was analyzed by flow cytometry. Mean ± SD, *n* = 3. **P < 0.01, vs. shCtrl. **C** DNA replication of MDA-MB-231 cells was analyzed by EDU staining. Mean ± SD, *n* = 3. **P < 0.01, vs. shCtrl. **D** Viability of MDA-MB-231 cells was analyzed by CCK8. Mean ± SD, *n* = 3. ***P < 0.001, vs. shCtrl. **E** EMP3, CCNE2, CDK2, P21, and PCNA expression in MCF7 cells was detected by western blot. **F** Cell cycle of MCF7 cells was analyzed by flow cytometry. Mean ± SD, *n* = 3. **P < 0.01, vs. OE Ctrl. **G** Viability of MCF7 cells was analyzed by CCK8. Mean ± SD, *n* = 3. ***P < 0.001, vs. OE Ctrl.

To verify whether EMP3 affects S-phase entry and DNA replication, MDA-MB-231 cells with high EMP3 abundance were transfected with EMP3 shRNA viruses to stably knock down EMP3. EMP3 knockdown upregulated CCNE2, CDK2, and PCNA and downregulated P21, a negative modulator of S-phage (Fig. 2A). Consistently, EMP3 knockdown increased the fraction of S-phase cells and the fraction of EDU-positive cells (Fig. 2B, C). Moreover, EMP3 knockdown increased cell viability (Fig. 2D). EMP3 knockdown in HS578T cells similarly promoted DNA replication and cell viability (Supplementary Fig. 2). Conversely, EMP3 overexpression in MCF7 cells which have a low level of EMP3 inhibited CCNE2, CDK2, and PCNA expression whereas induced P21 expression (Fig. 2E). Moreover, EMP3 overexpression decreased the fraction of S-phase cells and cell viability (Fig. 2F, G). Finally, the role of EMP3 in SK-BR-3 and BT474 cells which represent other subtypes of breast cancer was investigated. EMP3 overexpression similarly impeded S-phase entry and suppressed cell viability (Supplementary Fig. 3).

### EMP3 impairs DNA damage repair and enhances chemo-sensitivity

**Fig. 3:**
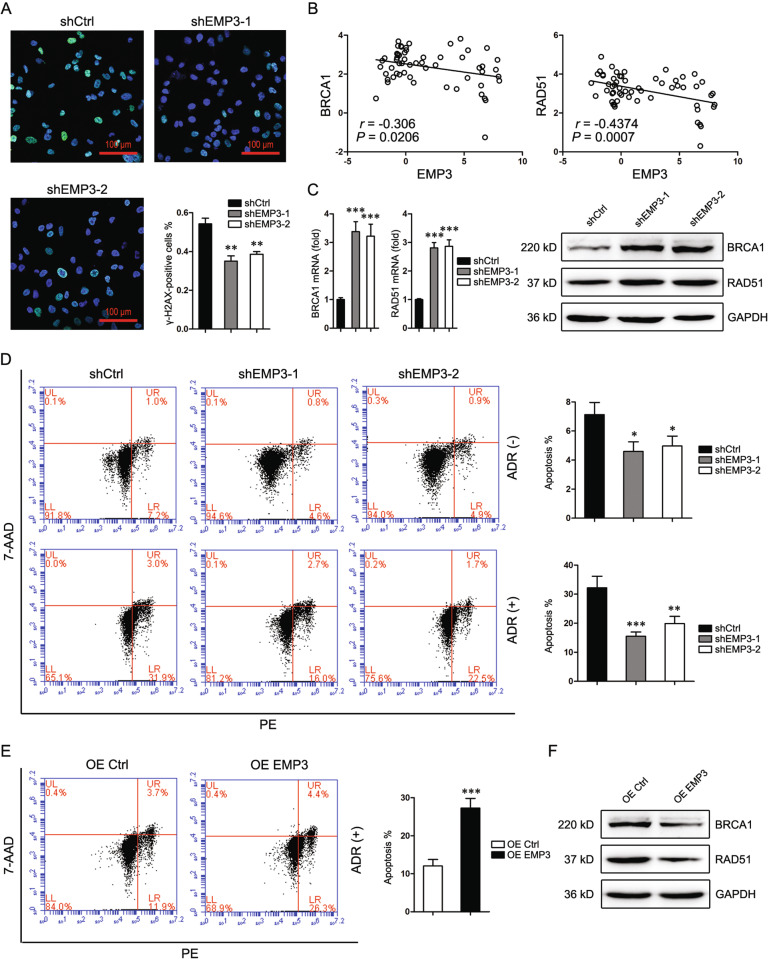
EMP3 impairs DNA damage repair and enhances chemo-sensitivity. **A** γ-H2AX expression in MDA-MB-231 cells was detected by Immunofluorescence staining 48 h after treatment with ADR (500 nM). Mean ± SD, *n* = 3. **P < 0.01, vs shCtrl. **B** CCLE database was interrogated for EMP3, BRCA1, and RAD51 expression. Correlation between two genes in a total of 57 breast cancer cell lines was analyzed by Pearson statistics. **C** BRCA1 and RAD51 expression in MDA-MB-231 cells were detected by western blot and real-time PCR. Mean ± SD, *n* = 3. ***P < 0.001, vs shCtrl. **D** Death of MDA-MB-231 cells was detected by flow cytometry 48 h after treatment with or without ADR (500 nM). Mean ± SD, *n* = 3. *P < 0.05, **P < 0.01, ***P < 0.001, vs. shCtrl. **E** Death of MCF7 cells was detected by flow cytometry 48 h after treatment with ADR (500 nM). Mean ± SD, *n* = 3. ***P < 0.001, vs. OE Ctrl. **F** BRCA1 and RAD51 expression in MCF7 cells were detected by western blot.

As DNA replication is closely related to DNA damage repair, the role of EMP3 in DNA damage repair was subsequently investigated. 48 h after treatment with Adriamycin (ADR) which induces DNA damage by inhibiting DNA topoisomerase II, EMP3-knockdowned MDA-MB-231 cells displayed less γ-H2AX staining compared to control cells, indicating that EMP3 knockdown promoted DNA damage repair (Fig. 3A). CCLE database interrogation revealed a negative correlation of EMP3 with BRCA1 and RAD51 (Fig. 3B). Consistently, EMP3 knockdown induced BRCA1 and RAD51 expression in both mRNA and protein levels (Fig. 3C). EMP3 knockdown slightly inhibited cell apoptosis, but significantly enhanced cell survival in the presence of ADR (Fig. 3D). EMP3 knockdown in HS578T cells also increased the capacity of DNA damage repair and the resistance to ADR (Supplementary Fig. 4). To the contrary, EMP3 overexpression sensitized MCF7 cells to ADR, accompanied by a downregulation of BRCA1 and RAD51 (Fig. 3E, F). These data indicate that EMP3 downregulation in breast cancer induces chemoresistance.

### EMP3 interferes with stem-like properties

**Fig. 4:**
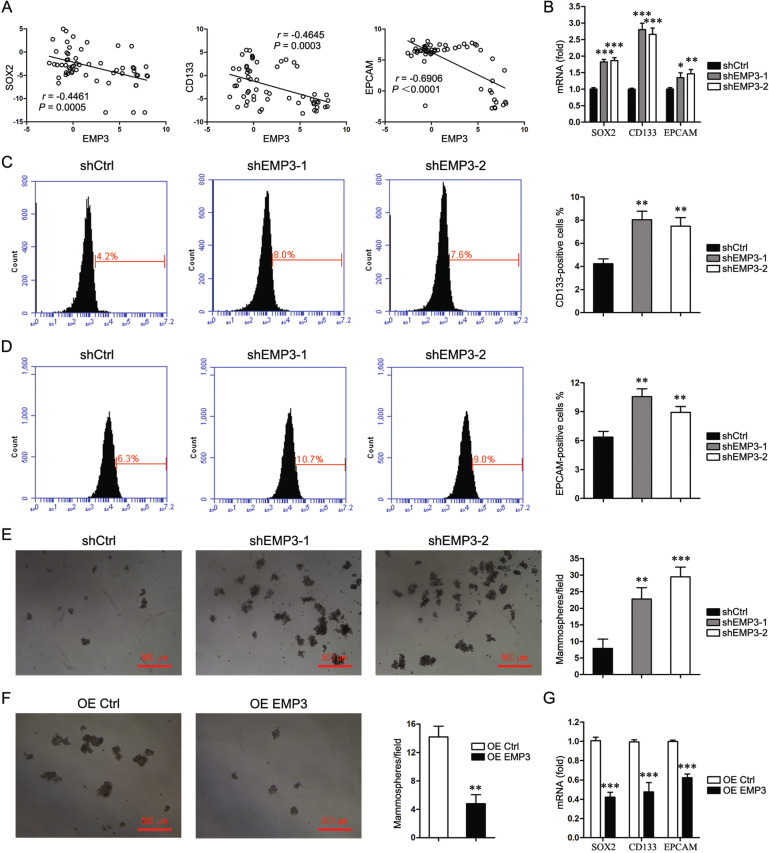
EMP3 interferes with stem-like properties. **A** CCLE database was interrogated for EMP3, SOX2, CD133, and EPCAM expression. Correlation between two genes in a total of 57 breast cancer cell lines was analyzed by Pearson statistics. **B** SOX2, CD133, and EPCAM expression in MDA-MB-231 cells were detected by real-time PCR. Mean ± SD, *n* = 3. *P < 0.05, **P < 0.01, ***P < 0.001, vs. shCtrl. **C** Fractions of CD133-positive MDA-MB-231 cells were detected by flow cytometry. Mean ± SD, *n* = 3. **P < 0.01, vs. shCtrl. **D** Fractions of EPCAM-positive MDA-MB-231 cells were detected by flow cytometry. Mean ± SD, *n* = 3. **P < 0.01, vs. shCtrl. **E** Mammosphere formation of MDA-MB-231 cells was shown. Mean ± SD, *n* = 3. **P < 0.01, ***P < 0.001, vs. shCtrl. **F** Mammosphere formation of MCF7 cells was shown. Mean ± SD, *n* = 3. **P < 0.01, vs. OE Ctrl. **G** SOX2, CD133, and EPCAM expression in MCF7 cells were detected by real-time PCR. Mean ± SD, *n* = 3. ***P < 0.001, vs. OE Ctrl.

Cancer stem cells are characterized by an increase in DNA damage repair capacity, therefore whether EMP3 modulates cancer cell stem-like properties were evaluated. Correlation analyses in the CCLE database uncovered a negative association of EMP3 with stem-related transcription factor SOX2 and stem-related markers CD133 and EPCAM (Fig. 4A). EMP3 knockdown upregulated SOX2, CD133, EPCAM expression in both MDA-MB-231 and HS578T cells (Fig. 4B and Supplementary Fig. 5A). Accordingly, EMP3 knockdown promoted the generation of CD133-positive cells and EPCAM-positive cells (Fig. 4C, D). Moreover, EMP3 knockdown induced mammosphere formation (Fig. 4E and Supplementary Fig. 5B). As expected, EMP3 overexpression in MCF7 cells inhibited mammosphere formation, and SOX2, CD133, and EPCAM expression (Fig. 4F, G). These data confirm that EMP3 hinders breast cancer cell stem-like properties.

### EMP3 inhibits Akt-mTor signaling and induces autophagy

**Fig. 5:**
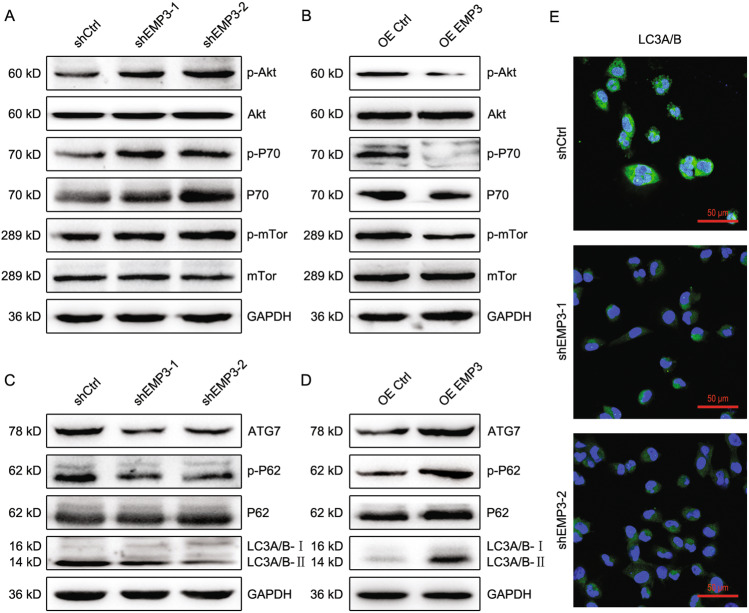
EMP3 inhibits Akt-mTor signaling and induces autophagy. **A** p-Akt/Akt, p-P70/P70, and p-mTor/mTor expression in MDA-MB-231 cells was detected by western blot. **B** p-Akt/Akt, p-P70/P70, and p-mTor/mTor expression in MCF7 cells were detected by western blot. **C** ATG7, p-P62/P62, and LC3A/B expression in MDA-MB-231 cells were detected by western blot. **D** ATG7, p-P62/P62, and LC3A/B expression in MCF7 cells were detected by western blot. **E** LC3A/B expression in MDA-MB-231 cells was detected by Immunofluorescence staining.

Intracellular signaling related to EMP3 was unknown in breast cancer. Akt-mTor signaling has a critical role in tumor cell growth and survival. Therefore, the effect of EMP3 on Akt-mTor signaling was investigated. EMP3 knockdown induced the phosphorylation of Akt, P70, and mTor in MDA-MB-231 cells; conversely, EMP3 overexpression suppressed the phosphorylation of these molecules, indicating that EMP3 interferes with the Akt-mTor pathway (Fig. 5A, B and Supplementary Fig. 6A, B). Given the association of Akt-mTor signaling with autophagy, whether EMP3 modulates autophagy was further assessed. EMP3 knockdown reduced expression of ATG7, phosphorylated P62, and LC3A/B-II, while EMP3 overexpression upregulated these proteins, demonstrating that EMP3 promotes breast cancer cell autophagy (Fig. 5C, D and Supplementary Fig. 6C, D). Finally, the regulation of EMP3 on LC3A/B expression was verified by Immunofluorescence staining (Fig. 5E).

### YTHDC1 is a downstream effector of EMP3

**Fig. 6:**
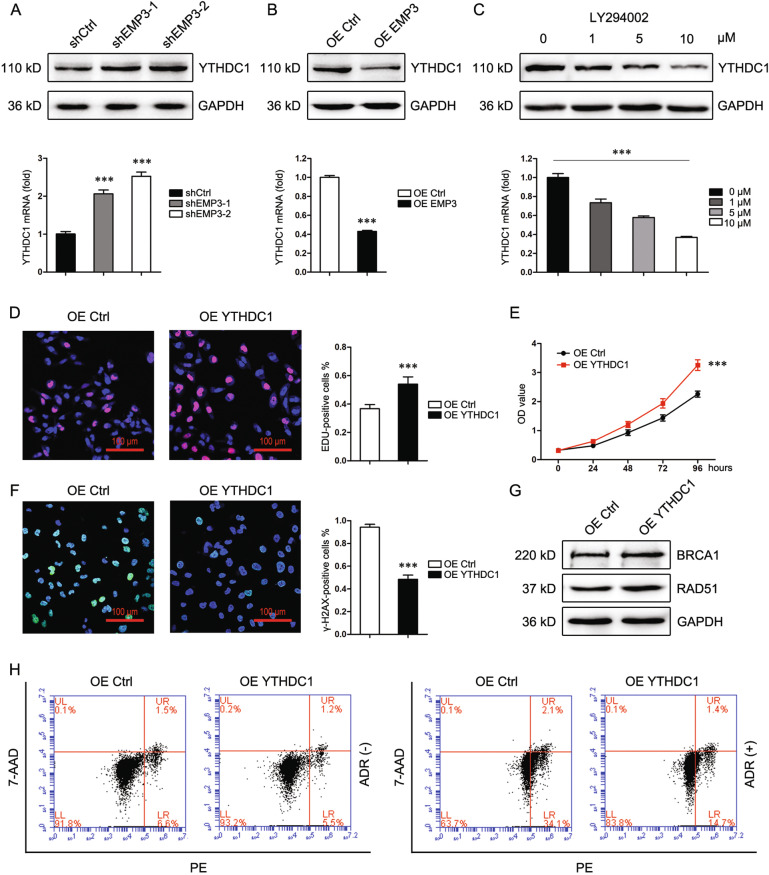
YTHDC1 is a downstream effector of EMP3. **A** YTHDC1 expression in MDA-MB-231 cells was detected by western blot and real-time PCR. ***P < 0.001, vs. shCtrl. **B** YTHDC1 expression in MCF7 cells was detected by western blot and real-time PCR. ***P < 0.001, vs. OE Ctrl. **C** YTHDC1 expression in MDA-MB-231 cells treated with LY294002 was detected by western blot and real-time PCR. ***P < 0.001. **D** DNA replication of MDA-MB-231 cells was analyzed by EDU staining. Mean ± SD, *n* = 3. ***P < 0.001, vs. OE Ctrl. **E** Viability of MDA-MB-231 cells was analyzed by CCK8. Mean ± SD, *n* = 3. ***P < 0.001, vs. OE Ctrl. **F** γ-H2AX expression in MDA-MB-231 cells was detected by Immunofluorescence staining 48 h after treatment with ADR (500 nM). Mean ± SD, *n* = 3. ***P < 0.001, vs OE Ctrl. **G** BRCA1 and RAD51 expression in MDA-MB-231 cells were detected by western blot. **H** Death of MDA-MB-231 cells was detected by flow cytometry 48 h after treatment with or without ADR (500 nM).

METTL3/YTHDC1-mediated m6A modification was recently shown involved in homologous recombination repair of DNA double-strand breaks [24]. Intriguingly, CCLE database interrogation revealed a negative correlation between EMP3 and YTHDC1 (Supplementary Fig. 7). Therefore, whether EMP3 modulates YTHDC1 was investigated. Just as expected, EMP3 knockdown in MDA-MB-231 cells upregulated YTHDC1 in both mRNA and protein levels (Fig. 6A). To the contrary, EMP3 overexpression in MCF7 cells downregulated YTHDC1 (Fig. 6B). Furthermore, treatment with Akt inhibitor LY294002 attenuated YTHDC1 expression in MCF7 cells, indicating that EMP3 modulates YTHDC1 at least in part through Akt signaling (Fig. 6C). Next, the effect of YTHDC1 on DNA replication and DNA damage repair was explored. YTHDC1 overexpression in MDA-MB-231 cells elevated the fraction of EDU-positive cells accompanied by an increase in cell viability (Fig. 6D, E). Cells with YTHDC1 overexpression exhibited less γ-H2AX staining and more BRCA1/RAD51 expression, compared to control cells (Fig. 6F, G). Consistently, YTHDC1 overexpression induced resistance to ADR (Fig. 6H). These results indicate that YTHDC1 promotes DNA replication and DNA damage repair.

## Discussion

Loss and gain of function studies have revealed that EMP3 impedes breast cancer cell S-phase entry, DNA replication, and proliferation. In combination with prognosis analysis, this finding shows that EMP3 functions as a suppressor of tumor growth in this type of cancer. EMP3 negatively modulates BRCA1 and RAD51, indicating that it suppresses homologous recombination repair of DNA double-strand breaks. This mechanism makes EMP3 have the capacity to sensitize cancer cells to DNA-damaging agents including some chemotherapeutic drugs and radiation. Therefore, EMP3 downregulation contributes to the chemoresistance and radioresistance of breast cancers.

As a negative regulator of stem-like properties, EMP3 inhibits the expansion of breast cancer stem cells. DNA damage repair is critical for the self-renewal of normal and cancer stem cells [25–27]. RAD51-associated protein 1 (RAD51AP1), which induces RAD51 activation, promotes tumor growth and chemoresistance by modulating the self-renewal of breast cancer stem cells [28]. This finding concurrently supports the role of RAD51 in maintaining the stem-like properties of breast cancer cells. Consistent with the fact that RAD51AP1 functions in both luminal ER-positive and triple-negative breast cancers, EMP3 affects RAD51 expression as well as DNA replication, DNA damage repair, and stem-like properties in both ER-positive MCF7 and triple-negative MDA-MB-231/HS578 cells.

Akt/mTOR signaling plays an important role in cell proliferation, cell growth, and cell survival. This pathway also regulates homologous recombination repair of DNA double-strand breaks [29]. EMP3 blocks Akt/mTOR pathway in breast cancer cells, suggesting that this pathway may mediate EMP3-induced suppression of DNA replication, cell growth, and DNA damage repair. Akt pathway was also shown to mediate CCR5-induced DNA damage repair in breast cancer stem cells [15]. Notably, EMP3 knockdown in melanoma C32 cells inhibited Akt phosphorylation and cell viability, indicating that the effect of EMP3 on Akt signaling and the role of EMP3 in malignancies are tumor-specific [30].

Recent studies have shown that methylation at the 6 position of adenosine (m^6^A) in RNA regulates DNA damage repair. Methyltransferase METTL3 is involved in DNA damage response to ultraviolet including nucleotide excision repair and trans-lesion synthesis [31]. Moreover, METTL3-mediated m6A modification and m6a reader YTHDC1 promote homologous recombination repair by modulating BRCA1 and RAD51 [24]. The negative modulation of YTHDC1 by EMP3 and the effect of YTHDC1 overexpression on breast cancer cells together demonstrate that YTHDC1 and m6A modification at least in part mediate EMP3-induced suppression of homologous recombination repair.

**Fig. 7:**
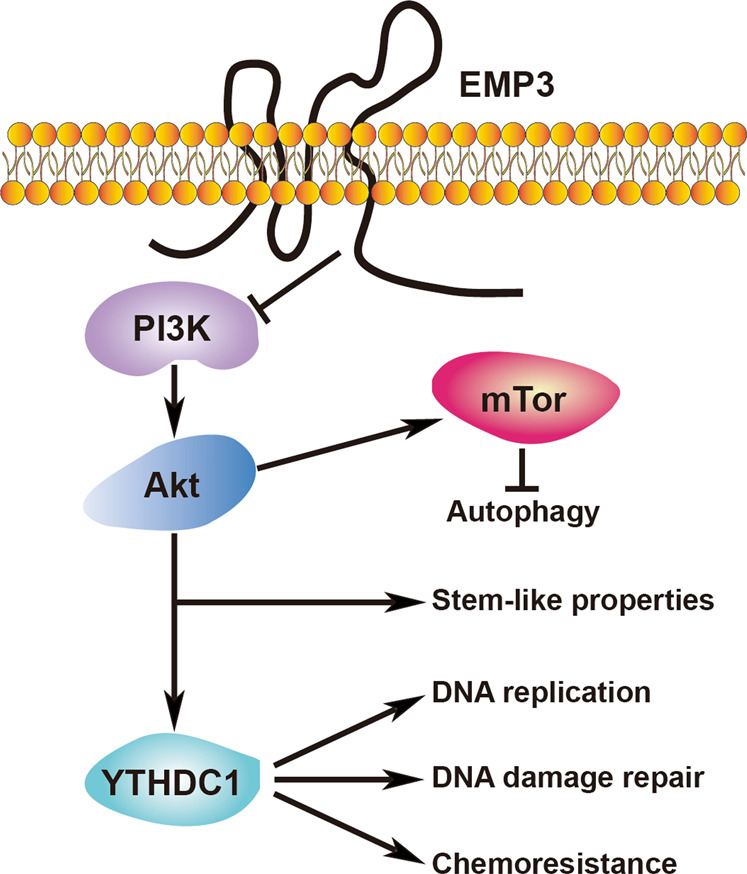
A schematic figure showing EMP3 signaling pathway. EMP3 regulates autophagy, stem-like properties, DNA replication, DNA damage repair and chemoresistance through blocking PI3K-Akt pathway. Modulation of DNA replication, DNA damage repair and chemoresistance by EMP3 is dependent on YTHDC1.

In summary, EMP3 for the first time was revealed to inhibit S-phase entry, DNA replication, DNA damage repair, chemotherapeutic drug resistance, stem-like properties, and Akt-mTOR signaling activation. EMP3 is not only a tumor suppressor but also a target to enhance chemosensitivity in breast cancer. A link between EMP3 and YTHDC1 hints at the involvement of m6A modification in EMP3-mediated DNA damage repair (Fig. 7).

## Materials and methods

### In silico analysis

The association of EMP3 with survival was analyzed using the TCGA database and Kaplan–Meier plotter website (http://kmplot.com/analysis/), respectively. Cancer Cell Line Encyclopedia (CCLE) database was interrogated for gene mRNA expression in a series of human breast cancer cell lines. Correlation between two genes was analyzed by Pearson statistics. GSEA was performed using the TCGA database. The gene pathways differentially expressed between EMP3-high and EMP3-low breast cancer samples were analyzed.

### Cell culture

MDA-MB-231, HS578T, MCF7, SK-BR-3, and BT474 cells were obtained from Nanjing KeyGen Biology (Nanjing, China). All cell lines have been authenticated using STR profiling. MDA-MB-231, HS578T, MCF7, and SK-BR-3 cells were cultured in DMEM (Hyclone) with 10% fetal bovine serum (FBS). BT474 cells were cultured in RPMI 1640 medium (Hyclone) with 10% FBS. All cells were cultured at 37 °C in a humidified incubator with 5% CO_2_. For γ-H2AX staining, cells were pretreated with ADR (Solarbio, China) at a final concentration of 500 nM for 24 h.

### Cell transfection

Cells were transfected with shEMP3 lentiviruses (GV112/hU6-MCS-CMV-Puromycin, Genechem, China) to stably knock down EMP3 expression. Puromycin (2 μg/ml, Sigma) was used to select cells 48 h after infection. The target sequences for shEMP3 is 5′-cgCCTTGATCTATGCCATTCA-3′ and 5′-ccTTCACATCCTCATTCTTAT-3′. Cells were transfected with EMP3 and YTHDC1 overexpression plasmids (GV219/CMV-MCS-SV40-Neomycin, Genechem, China), respectively, using Lipofectamine 3000 in Opti-MEM medium according to the instructions.

### Western blot

Cells were washed three times with PBS and lysed on ice using RIPA lysis buffer with 1% PMSF, followed by centrifugation at 12,000 × *g* for 40 min at 4 °C. Protein concentrations were determined by the BCA kit. An equal amount of protein was resolved by SDS-PAGE and transferred to PVDF membranes. The membranes were blocked in 5% skimmed milk or 5% BSA at room temperature and incubated with primary antibodies at 4 °C overnight, followed by incubation with secondary antibodies conjugated with HRP at room temperature for 2 h. Target proteins were detected using a chemiluminescence detection kit. The primary antibodies are as follows: EMP3 (abcam, #ab73151, UK, 1:1000), CDK2 (Cell Signaling Technology, #2546, USA, 1:1000), Cyclin E2 (Cell Signaling Technology, #4132, USA, 1:1000), P21 (Cell Signaling Technology, #2947, USA, 1:1000), PCNA (SANTA, sc-71858, USA, 1:1000), BRCA1 (Cell Signaling Technology, #9010, USA, 1:1000), Survivin (ThermoFisher, PA5-16859, USA, 1:1000), p-Akt (Cell Signaling Technology, #9271, USA, 1:1000), Akt (Cell Signaling Technology, #9272, USA, 1:1000), p-P70 (Cell Signaling Technology, #9205, USA, 1:1000), P70 (Cell Signaling Technology, #9202, USA, 1:1000), p-mTOR (Cell Signaling Technology, #2971, USA, 1:1000), mTOR (Cell Signaling Technology, #2972, USA, 1:1000), ATG7 (Cell Signaling Technology, #8558, USA, 1:1000), p-P62 (Cell Signaling Technology, #13121, USA, 1:1000), P62 (Cell Signaling Technology, #8025, USA, 1:1000), LC3A/B (Cell Signaling Technology, #12741, USA, 1:1000), YTHDC1 (Cell Signaling Technology, #81504, USA, 1:1000) and RAD51 (abcam, #ab133534, UK, 1:1000).

### Cell cycle assay

The attached cells were washed twice with precooling PBS and harvested by trypsin without EDTA. The cell concentration was adjusted to 1 × 10^6^/ml. Then the cells were fixed with 70% ethanol overnight and washed twice with precooling PBS. 500 μl PI/RNaSeA staining solution was added at room temperature for 60 min in dark. Cell cycle distribution was analyzed by a FACS Calibur flow cytometer (BD).

### EDU staining

5 × 10^5^ cells were seeded on glass coverslips in 24-well plates for 24 h. After incubation with 100 μl EDU (50 μM, Ribobio, China) for 2 h, cells were fixed with 50 μl 4% paraformaldehyde for 30 min at room temperature and washed with Glycine solution (2 mg/ml) once and with PBS twice. Then cells were incubated with a penetrating agent (0.5% Triton X-100 in PBS) and washed with PBS once. Thereafter, 100 μl 1 × Apollo® staining solution was added into each well followed by incubation at room temperature for 30 min in dark. Finally, 100 μl 1 × Hoechst 33342 reaction solution was added to dye the DNA for 30 min in dark. A laser scanning confocal focus microscope was used to visualize the staining. Positive cells were counted in random five fields.

### Cell viability assay

Cells were incubated in 96 well plates with 100 μl culture medium in each well (MDA-MB-231: 5 × 10^3^ cells/well; HS578T: 2 × 10^3^ cells/well; MCF7: 8 × 10^2^ cells/well; SK-BR-3: 1 × 10^3^ cells/well; BT474: 2 × 10^3^ cells/well). After 24 h, cells were treated with a 10 μl Cell Counting Kit-8 (CCK8, Dojindo) reagent and incubated in an incubator at 37 °C. Finally, the absorbance value at 450 nm was detected at different time points using a microplate reader (Bio-Rad Laboratories, USA).

### Immunofluorescence

Cells were seeded on slides in a 24-well plate (MDA-MB-231: 5 × 10^5^/well; HS578T: 4 × 10^5^/well) and grown in an incubator at 37 °C for 24 h. Then cells were fixed with paraformaldehyde for 20 min, washed with PBS three times, and permeabilized with 0.5% Triton X-100 for 10 min at room temperature. The slides were blocked in 5% donkey serum with 0.3% Triton X-100 in PBS at room temperature for 1 h and incubated with γ-H2AX (Cell Signaling Technology, #9718, USA, 1:400) or LC3A/B (Cell Signaling Technology, #12741, USA, 1:100) at 4 °C overnight. Subsequently, the slides were incubated with Alexa Fluor 488-conjugated anti-mouse IgG for 2 h, counterstained with DAPI in dark, and visualized using a laser scanning confocal focus microscope.

### Real-time PCR

Total RNA was extracted from cells with RNAiso Plus (Takala). The cDNA was synthesized from 1 μg of total RNA using PrimeScript RT reagent Kit containing gDNA Eraser (Takala). Real-time PCR was performed on ABI PRISM 7300 Sequence Detection system (Applied Biosystems, USA) with TB Green™ Premix Ex Taq II (Takala). 2^−ΔΔ^ Ct method was used to analyze the gene expression. The sequences were listed in Table 1.

**Table 1:** Primers for real-time PCR.

Genes	Primers (5′–3′)
EMP3-forward	AAGATCAGTACCTCTCAGATGG
EMP3-reverse	GCAGCACAAGAGACGTATCATA
EXO1-forward	GCTCGGCTAGGAATGTGCAGAC
EXO1-reverse	CCCACGCAGTGATGACAGGTAG
RFC4-forward	AAACCACCCGATTCTGTCTTAT
RFC4-reverse	CTTGGCAATGTCTAGTAATCGC
POLE2-forward	GTCTTAGCAGAAGGTTGGTTTG
POLE2-reverse	TGCAGAAGTCTTCACGAGTGTA
EPCAM-forward	GTCTGTGAAAACTACAAGCTGG
EPCAM-reverse	CAGTATTTTGTGCACCAACTGA
CD133-forward	GTGGCGTGTGCGGCTATGAC
CD133-reverse	CCAACTCCAACCATGAGGAAGACG
YTHDC1-forward	TGGATGATTTCCTTCGTCGCACAC
YTHDC1-reverse	TCACGTCCTCTATCTCGCTCTCTG
RAD51-forward	TGGCAGTGGCTGAGAGGTATGG
RAD51-reverse	GGTCTGGTGGTCTGTGTTGAACG
SOX1-forward	ACATGAACGGCTGGAGCAACG
SOX1-reverse	CTGCGAGCTGGTCATGGAGTTG
GAPDH-forward	CAGGAGGCATTGCTGATGAT
GAPDH-reverse	GAAGGCTGGGGCTCATTT

### Apoptosis assay

Cells were digested with trypsin without EDTA and washed with PBS twice. 1 × 10^5^ cells were re-suspended in a 500 μl Binding Buffer (KeyGEN BioTECH, China). Subsequently, 5 μl Annexin V-PE and 5 μl 7-AAD solution (KeyGEN BioTECH, China) were added for 15 min in dark. The samples were analyzed by a FACS Accuri C6 PLUS (BD).

### Cell subpopulation assay

Cells were washed with Straining buffer (Invitrogen) twice and re-suspended with 500 μl Straining buffer. Then cells were incubated with 5 μl CD133 antibody (Invitrogen, 17-1338-42, USA) or EPCAM antibody (Invitrogen, 12-9326-42, USA) on ice for 40 min in dark. Subsequently, cells were re-suspended with a 500 μl Straining Buffer (Invitrogen) and tested on the FACS Accuri C6 PLUS (BD).

### Tumorisphere formation

Cells were seeded in a six-well low-attachment surface polystyrene culture plate (Corning Costar, USA, MDA-MB-231: 8 × 10^3^ cells/well; HS578T: 1 × 10^4^ cells/well; MCF7: 3 × 10^3^ cells/well) and incubated with complete MammoCult™ Human Medium (STEMCELL Technologies, USA) in an incubator at 37 °C for 7 days. Spheroids were counted in randomly selected five fields.

### Statistical analysis

All experiments were performed in triplicate. Data were presented as mean ± SD and analyzed using GraphPad Prism 8 software. Differences were analyzed by two-sided Student’s *t*-test, one-way or two-way ANOVA. *P*-value < 0.05 was considered significant.

## Supplementary information

Supplementary Figure Legends

Supplementary Figure 1

Supplementary Figure 2

Supplementary Figure 3

Supplementary Figure 4

Supplementary Figure 5

Supplementary Figure 6

Supplementary Figure 7

## Data Availability

The published article includes all data sets generated/analyzed for this study.
