# Supplementary material for: EMP3 negatively modulates breast cancer cell DNA replication, DNA damage repair, and stem-like properties
Source: Cell Death Dis. 2021 Sep 12;12(9):844. doi: 10.1038/s41419-021-04140-6 (PMC8435533; doi:10.1038/s41419-021-04140-6)
Supplement: Supplementary file 1 — Supplementary Figure Legends [file 41419_2021_4140_MOESM1_ESM.docx]

**Supplementary Figure legends**

**Supplementary Fig. 1** The association of EMP3 with OS (**A**), RFS (**B**) and DMFS (**C**) was analyzed in Kaplan–Meier plotter website.

**Supplementary Fig. 2 A** Cell cycle of HS578T cells was analyzed by flow cytometry. Mean ± SD, n = 3. ****P* < 0.001, vs. shCtrl. **B** Viability of HS578T cells was analyzed by CCK8. Mean ± SD, n = 3. ****P* < 0.001, vs. shCtrl.

**Supplementary Fig. 3 A** Cell cycle of SK-BR-3 cells was analyzed by flow cytometry. Mean ± SD, n = 3. ****P* < 0.001, vs. OE Ctrl. **B** Viability of SK-BR-3 cells was analyzed by CCK8. Mean ± SD, n = 3. ****P*< 0.001, vs. OE Ctrl. **C** Cell cycle of BT474 cells was analyzed by flow cytometry. Mean ± SD, n = 3. ***P* < 0.01, vs. OE Ctrl. **D** Viability of BT474 cells was analyzed by CCK8. Mean ± SD, n = 3. ****P*< 0.001, vs. OE Ctrl.

**Supplementary Fig. 4 A** γ-H2AX expression in HS578T cells was detected by Immunofluorescence staining 48 hours after treatment with ADR (500 nM). Mean±SD, n = 3. ****P* < 0.001. **B** Death of 578T cells was detected by flow cytometry 48 hours after treatment with or without ADR (500 nM). Mean ± SD, n = 3. **P* < 0.05, ****P* < 0.001, vs. shCtrl.

**Supplementary Fig. 5 A** SOX2, CD133 and EPCAM expression in HS578T cells was detected by Real-time PCR. Mean ± SD, n = 3. ***P* < 0.01, ****P* < 0.001, vs. shCtrl. **B** Mammosphere formation of HS578T cells was shown. Mean ± SD, n = 3. ****P* < 0.001, vs. shCtrl.

**Supplementary Fig. 6** The relative expression of phosphorylated protein in relation to the total protein. Mean ± SD, n = 3. ***P* < 0.01, ****P* < 0.001, vs. shCtrl; ^###^*P* < 0.001, vs. OE Ctrl.

**Supplementary Fig. 7** CCLE database was interrogated for EMP3 and YTHDC1 expression. Correlation between these two genes in a total of 57 breast cancer cell lines was analyzed by Pearson statistics.
