# Supplementary figures and images for: EMP3 negatively modulates breast cancer cell DNA replication, DNA damage repair, and stem-like properties
Source: Cell Death Dis. 2021 Sep 12;12(9):844. doi: 10.1038/s41419-021-04140-6 (PMC8435533; doi:10.1038/s41419-021-04140-6)

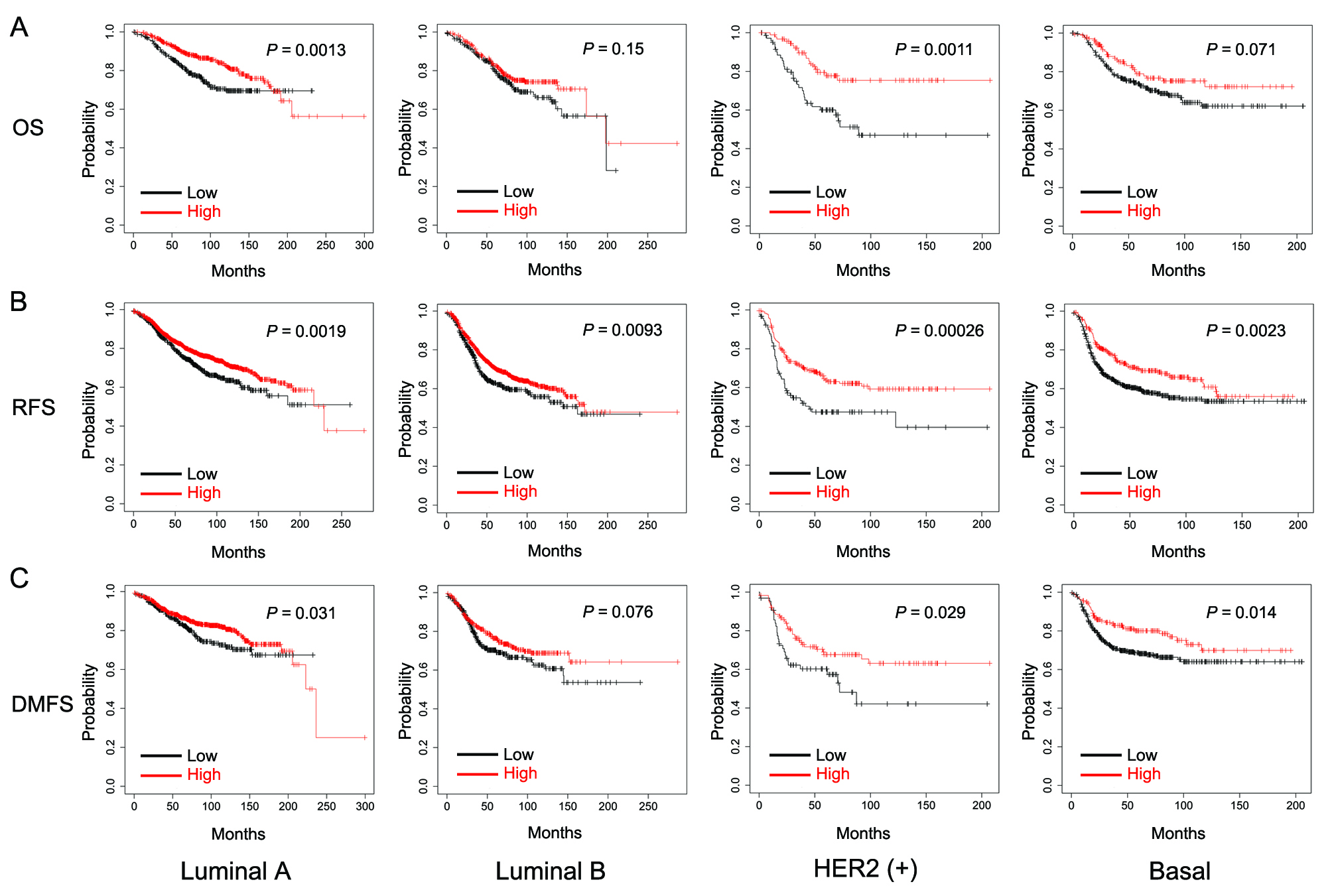

Supplement: Supplementary file 2 — Supplementary Figure 1 [file 41419_2021_4140_MOESM2_ESM.jpg]

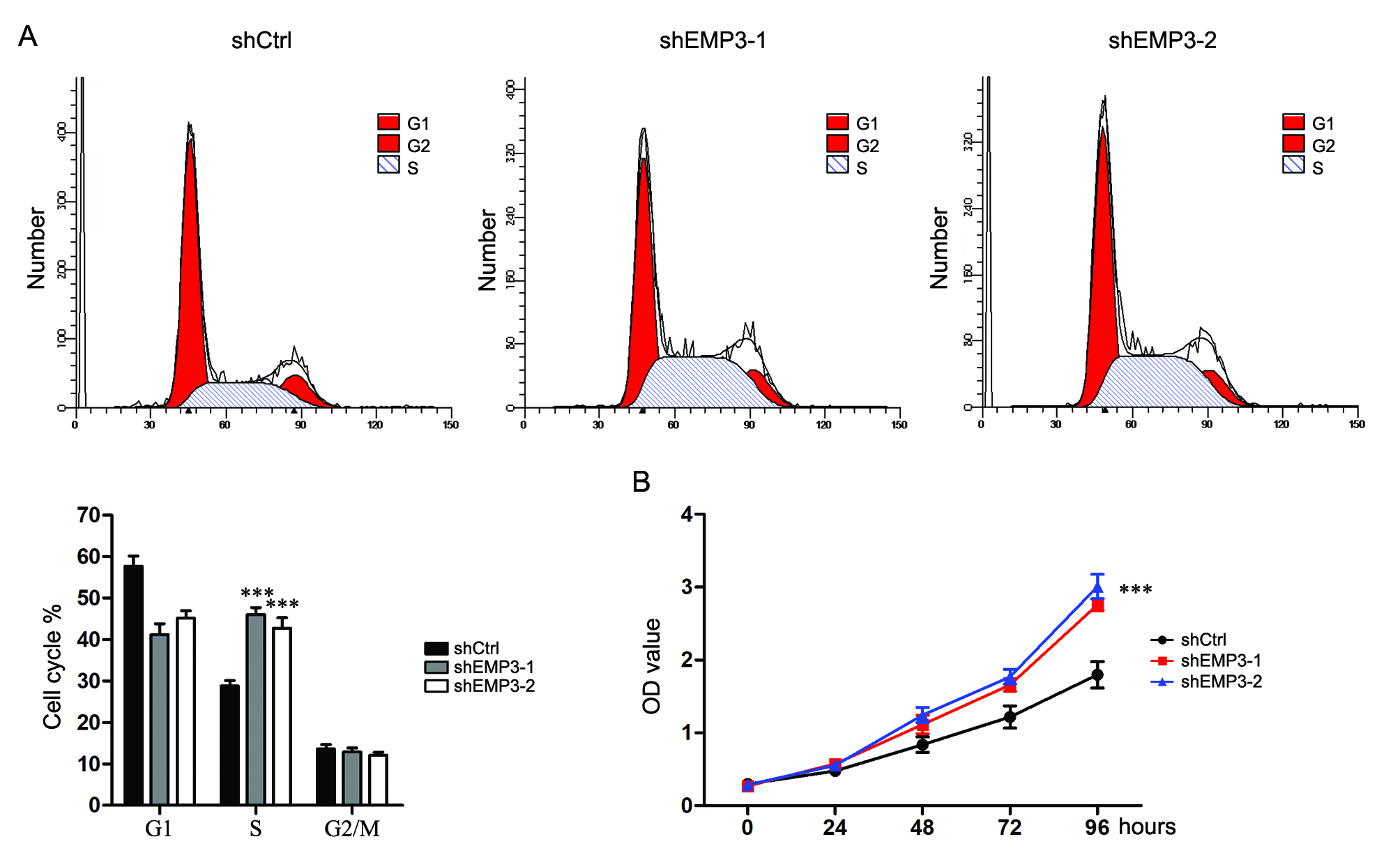

Supplement: Supplementary file 3 — Supplementary Figure 2 [file 41419_2021_4140_MOESM3_ESM.jpg]

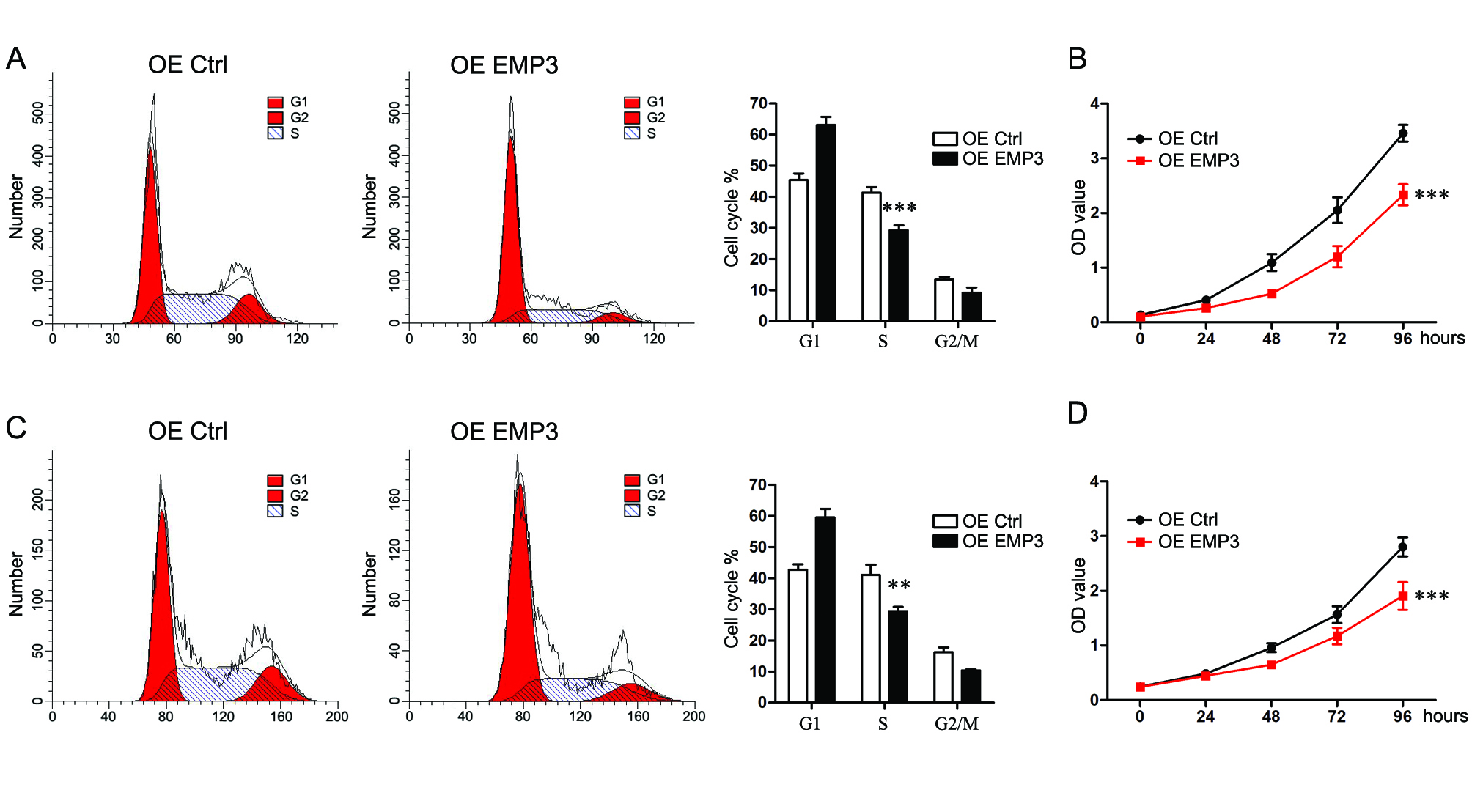

Supplement: Supplementary file 4 — Supplementary Figure 3 [file 41419_2021_4140_MOESM4_ESM.jpg]

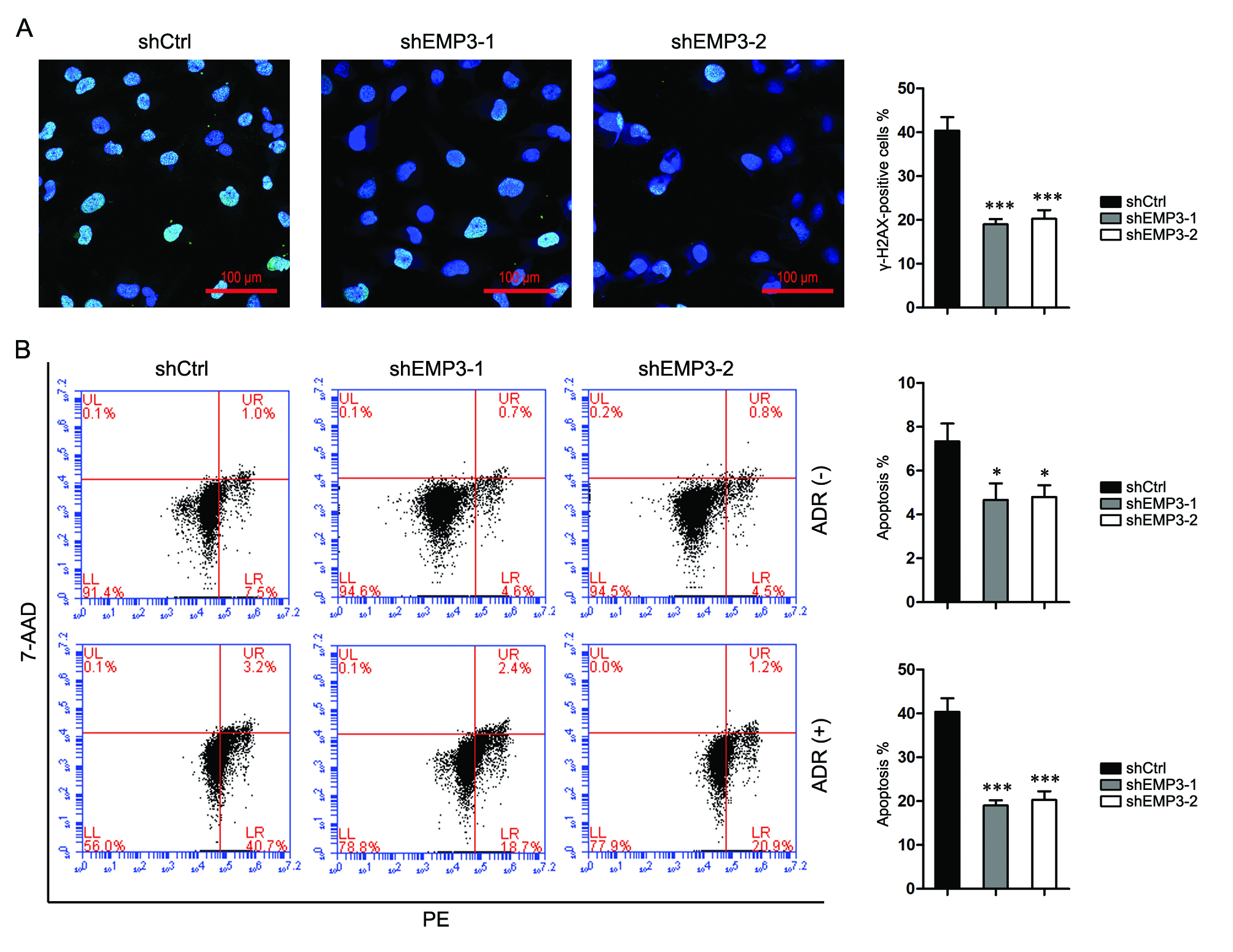

Supplement: Supplementary file 5 — Supplementary Figure 4 [file 41419_2021_4140_MOESM5_ESM.jpg]

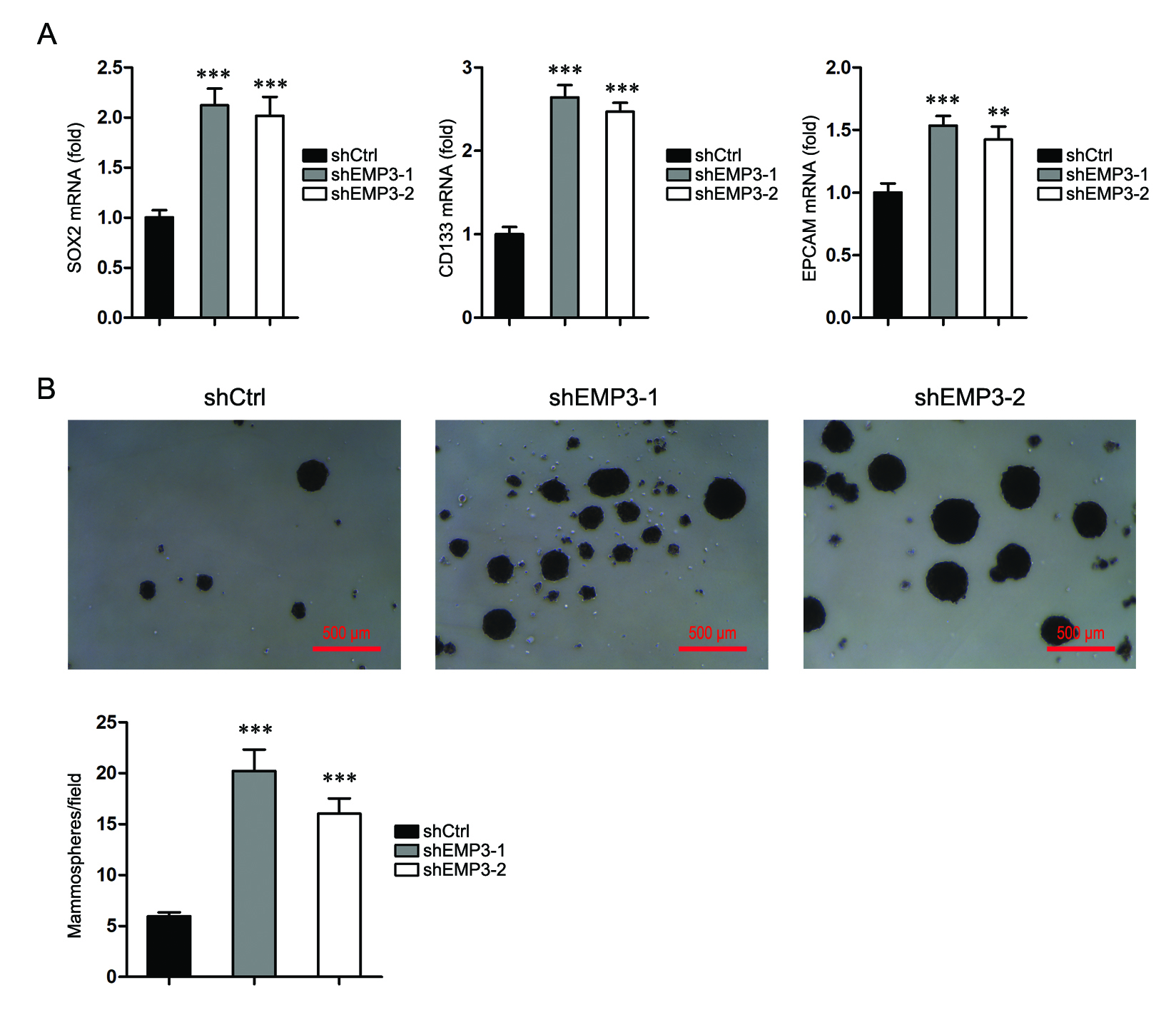

Supplement: Supplementary file 6 — Supplementary Figure 5 [file 41419_2021_4140_MOESM6_ESM.jpg]

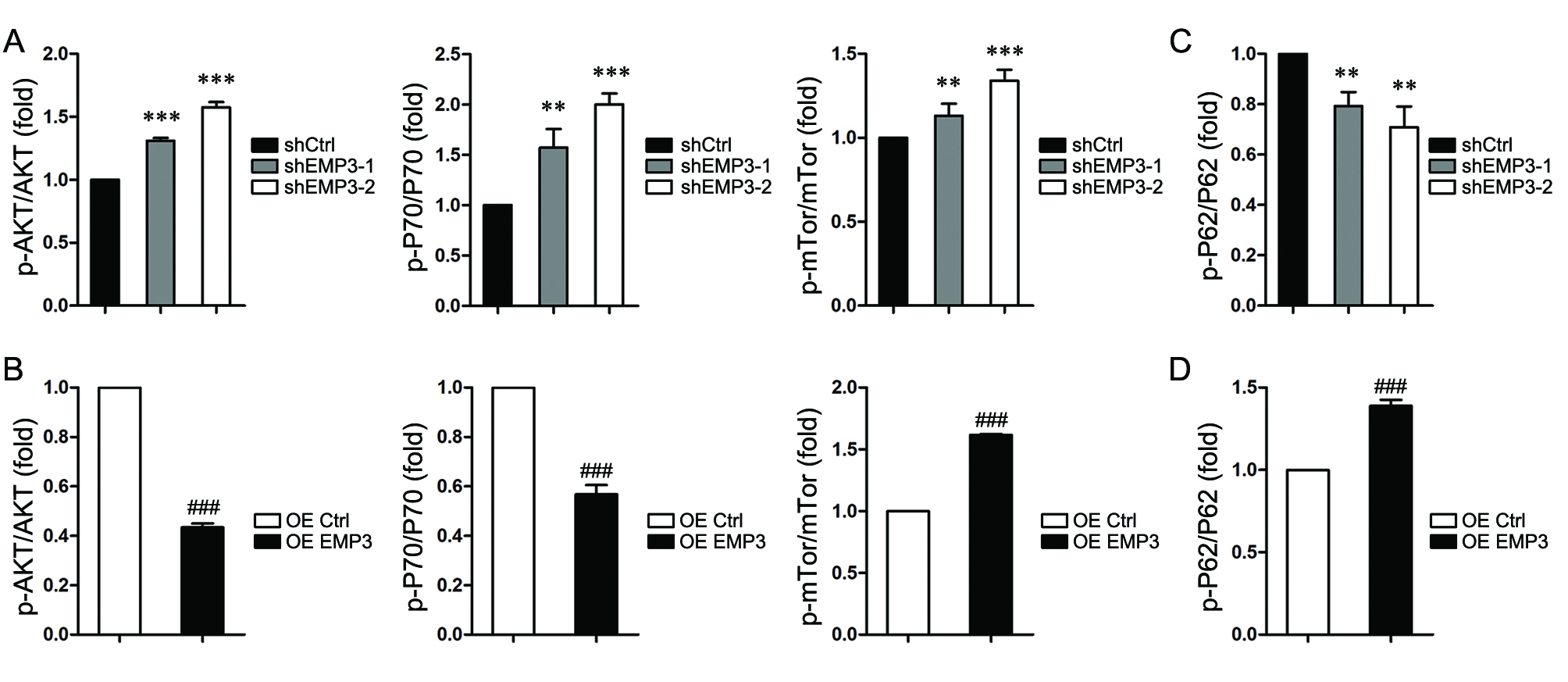

Supplement: Supplementary file 7 — Supplementary Figure 6 [file 41419_2021_4140_MOESM7_ESM.jpg]

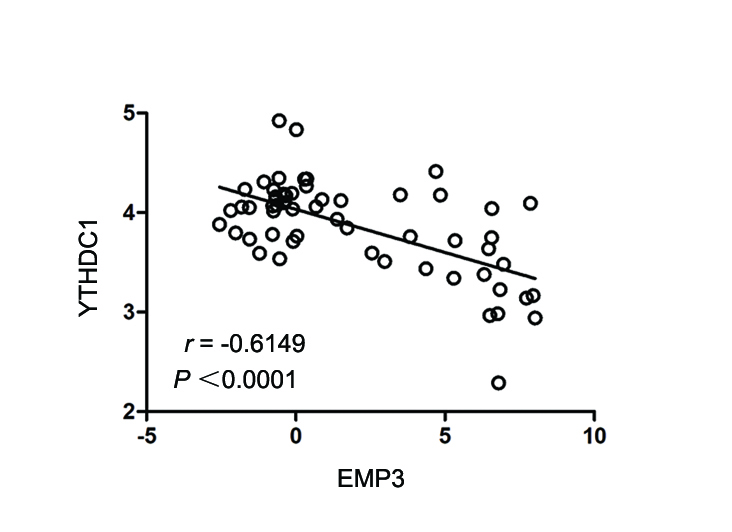

Supplement: Supplementary file 8 — Supplementary Figure 7 [file 41419_2021_4140_MOESM8_ESM.jpg]
